# 2026 American Society for Microbiology Awards and Prize Program: honorees from clinical microbiology

**DOI:** 10.1128/jcm.00001-26

**Published:** 2026-02-17

**Authors:** Erik Munson

**Affiliations:** 1 Wisconsin Clinical Laboratory Network Laboratory Technical Advisory Group 5505, Madison, Wisconsin, USA; Vanderbilt University Medical, Nashville, Tennessee, USA

**Keywords:** ASM Awards and Prize Program

## EDITORIAL

In September 2025, the American Society for Microbiology (ASM) announced recipients of honors in the context of the ASM Awards and Prize Program for calendar year 2026. A total of 17 awards, cast across multiple disciplines of the microbial sciences, as well as the ASM Microbiome Data Prize, have collectively been awarded on an annual basis since 2024—with a history of the Awards program dating back to 1968 (ASM Carski Award for Undergraduate Education). The American Academy of Microbiology (AAM), an honorific leadership group and scientific expert panel within ASM, administers this program that highlights outstanding research and/or contributions at every career level and subdiscipline of the microbial sciences. A complete listing of Award honorees for 2026 can be found with the following link: https://asm.org/press-releases/2025/september/asm-announces-honorees-for-2026-awards-and-prize-p.

Past roll calls of ASM Awards and Prize Program recipients relative to the clinical microbiology discipline have traditionally included honorees for the ASM Scherago-Rubin Award for Clinical Microbiology, the ASM Award for Research or Leadership in Clinical Microbiology, and (founded in 2024) the ASM Award for Early Career Clinical Microbiology Research. It is not imperative that the ASM Award for Service be bestowed upon a clinical microbiologist, as the award is designed to recognize an individual for outstanding contributions through service to the microbiological community on behalf of ASM. Yet for the sixth consecutive year (1–5), the recipient of this award comes from our clinical microbiology discipline. Recipients of three of the four aforementioned Awards will be featured in this Editorial.

The fourth awardee, Amy Mathers, M.D., D(ABMM), Bayer Corporation-Gerald L. Mandell Endowed Chair of Medicine at the University of Virginia and winner of the ASM Award for Research or Leadership in Clinical Microbiology, will be the subject of a biographical feature that will be published in a later edition of the *Journal of Clinical Microbiology*. Lastly, apart from the ASM Awards and Prize Program, the AAM annually selects an expert microbiologist to present a lecture at the ASM Microbe meeting under the auspices of the Elizabeth O. King Lecturer Award. This award seeks to promote the translation of science into solutions to improve public health and to inspire additional research into public health and the discovery of new pathogens. The award is supported by the New York Community Trust-Audrey Rheinstrom and Anne Blevins Fund. This year’s Lecturer, Carey-Ann Burnham, Ph.D., D(ABMM) from Pattern Bioscience in Austin, Texas, is no stranger to the pages of ASM Journals, now serving as editor-in-chief of *ASM Case Reports* (6). Dr. Burnham was also presented with the ASM Award for Research or Leadership in Clinical Microbiology in 2020; the reader is referred to a brief summary of her early career trajectory in a previous editorial (7) corresponding to that Award.

The ASM Scherago-Rubin Award for Clinical Microbiology was established by the late clinical microbiologist Dr. Sally Jo Rubin in honor of her grandfather, Morris Scherago. The award recognizes exceptional accomplishments of a non-doctoral-level, bench technologist who is involved in routine diagnostic work. Nominees (and awardees) are expected to demonstrate a strong record of engagement in microbiology venues outside of their primary vocation. These can include ASM or collaboratives with a significant number of clinical microbiologists. Mr. Idzi Potters, B.Sc., M.L.T., and a parasitology laboratory technician at the Institute of Tropical Medicine in Antwerp, Belgium, is the 2026 recipient of the ASM Scherago-Rubin Award for Clinical Microbiology. Mr. Potters received an undergraduate degree in biochemistry at Karel de Grote Hogeschool campus in Hoboken, Belgium. He later received a second degree in biomedical laboratory technology from the same institution. Mr. Potters has been employed at the Institute of Tropical Medicine since 1997, the first 4 years of which were devoted to the Central Laboratory for Clinical Biology and the remainder in his current position in the parasitology clinical reference laboratory. He also participates in the formal education of postgraduate students, medical students, and residents at the Institute. Mr. Potters’s personal statement notes, “I find it an immense advantage to be able to combine teaching and laboratory work: teaching stimulates me to look things up to stay up to date in the laboratory, and conversely, I can speak from experience during courses because I spend most of my time in the laboratory, so I know what I’m talking about during my lectures.”

In her primary nomination letter for the Award, Dr. Bobbi Pritt (4) of the Mayo Clinic in Rochester, Minnesota, deemed Mr. Potters as “an expert in clinical parasitology—a small and specialized field (8, 9). He spends the majority of his time in the laboratory examining clinical specimens for parasites and also participates in educational and humanitarian efforts at the institutional, national, and international levels.” Of note, Mr. Potters founded a *Schistosoma* laboratory in Richard-Toll, Senegal (10) that provides training and background knowledge for medical professionals. In addition, he organized a malaria “train the trainers” event in the Democratic Republic of the Congo that focused on advanced microscopy. Dr. Pritt proceeded to recount written testimonies by a pair of Mr. Potter’s colleagues in Belgium who view him as a “go-to” at the international scene for complex parasitology microscopy, further noting that he is one of just two members of an informal international parasitology interest group who do not possess an M.D. and/or Ph.D. degree. In-depth knowledge of parasite morphology, life cycle, clinical presentation, and epidemiology is apparently not enough for Mr. Potters, as his colleagues note that he is in the process of teaching himself the histopathology of parasitic infections to expand the scope of his expertise.

Mr. Potters readily contributes to scholarly activity outside of the diagnostic laboratory. He is credited with 15 peer-reviewed publications in the primary literature. He is also responsible for important guidance documents, including those published by the World Health Organization (WHO) (11), as well as past (12) and in-process chapters for the *Manual of Clinical Microbiology*. Mr. Potters shares a long-standing commitment toward both advancement and raising visibility of the diagnostic parasitology field, evidenced by participation in the European Society for Clinical Microbiology and Infectious Disease Study Group for Clinical Parasitology, the WHO Diagnostic and Technical Advisory Group for Neglected Tropical Diseases, and the ever-famous “Creepy Dreadful Wonderful Parasites” blog hosted by Dr. Pritt. Mr. Potters himself stated, “my work is more than just a job—it is my passion” and perhaps has taken diagnostic parasitology to the next level in reporting, “my 12-year-old son thinks it’s great that I work with parasites (he’s crazy about worms), so I try to involve him as much as possible. With a bit of luck, he will become a worm specialist too…we can only hope.”

Michael J. Loeffelholz, Ph.D., D(ABMM), currently Vice President, Scientific Affairs at Cepheid in Sunnyvale, California, is the 2026 recipient of the ASM Award for Service. Nominees (and awardees) for the Award are expected to demonstrate meaningful service to ASM and its members in the context of editorial board membership, workshop leader, meeting organizer, and/or service on committees. Such volunteerism extends multiple years and does not involve serving on the ASM Board of Directors nor holding the office of ASM President, Secretary, or Treasurer. Dr. Loeffelholz has logged extensive experience in the fields of industry, academia, public health, and clinical diagnostics, which has translated well into means to promote ASM as a scientific community.

Dr. Loeffelholz earned his undergraduate degree in microbiology at the University of Iowa. His doctoral work on *Acinetobacter calcoaceticus* was completed at the University of Ohio, followed by a postdoctoral stint at Diagnostic Hybrids, Incorporated, also located in Athens, Ohio. A postdoctoral fellowship in the AAM-accredited residency program in medical and public health laboratory microbiology was completed at the University of Rochester in Rochester, New York. Stops in industry (prior to his current position) include those with Roche Molecular Systems, during which time the first U.S. Food and Drug Administration-cleared PCR-based test for infectious disease diagnostics (*Chlamydia trachomatis*) was developed (13). State public health laboratory microbiology directorships were assumed in Arkansas and Iowa, with the latter resulting in two publications elucidating the performance of *Bordetella pertussis* nucleic acid amplification testing relative to both other diagnostic modalities and potentially cross-reactive *Bordetella* spp (14, 15). Clinical laboratory directorships in Ohio and Minnesota preceded a directorship/faculty position at the University of Texas Medical Branch, Galveston, from 2008 to 2018. Dr. Loeffelholz also directed the ASM Committee on Postgraduate Educational Programs (CPEP)-approved fellowship program during that time.

One of Dr. Loeffelholz’s trainees, Dr. Daniel Ortiz of Beaumont Health in Royal Oak, Michigan, provided primary nomination for the award. Dr. Ortiz wrote, “Dr. Loeffelholz embodies the spirit of this award through a remarkable lifetime of sustained and selfless commitment to the ASM community and the field of clinical microbiology…his impact is evident across ASM committees, journals, certification boards, and training programs…his influence on diagnostic standards, assay validation, and laboratory best practices (16) is nationally recognized.” Relative to ASM, Dr. Loeffelholz served as a member of the American College of Microbiology, American Board of Medical Microbiology Examination Development Committee from 2002 to 2009, member of the ASM Division C Chair Nominating Committee in 2010, editor of *Journal of Clinical Microbiology* from 2010 to 2021, editorial board member of *Clinical and Vaccine Immunology* in 2016 and 2017, member of the ASM Professional Affairs Committee from 2012 to 2021, and was appointed as a member of the ASM Council on Microbial Sciences in 2016, serving until 2018. Dr. Loeffelholz’s service has extended to entities such as Clinical and Laboratory Standards Institute (including documents MM06-A and M41-A; Board of Director membership since 2024; Expert Panel on Point-of-care Testing membership since 2019), the Pan American Society for Clinical Virology (including election as Secretary/Treasurer), the Association of Public Health Laboratories (including participation on a working group for development of Laboratory Guidelines for Syphilis Serology), and the U.S. Centers for Disease Control and Prevention (including an 8-year membership on the Infectious Disease Laboratory Working Group).

Beyond the 90 peer-reviewed publications, 21 book chapters, and items previously mentioned throughout this discourse, Dr. Loeffelholz believes his most significant act of service pertains to “training and mentoring the next generation of leaders in microbial sciences.” Fruits of such labor are exemplified in the Foreword that Dr. Loeffelholz was requested to scribe for the ASM Press/Wiley publication entitled *101 Topics for Clinical Microbiology Laboratory Leaders: Accreditation, Verification, Quality Systems, and More* (17).

The ASM Award for Early Career Clinical Microbiology Research has been bestowed upon Kyle Rodino, Ph.D., D(ABMM), Assistant Professor of Clinical Pathology and Laboratory Medicine at the University of Pennsylvania School of Medicine; Assistant Director of the Clinical Microbiology Laboratory and Director of the Rittenhouse Molecular Laboratory at the Hospital of the University of Pennsylvania—all in Philadelphia, Pennsylvania. Much like his primary nominator for the Award, Dr. Melissa Miller (1) of the University of North Carolina at Chapel Hill, North Carolina, Dr. Rodino completed an undergraduate degree in Clinical Laboratory Science (formerly known as Medical Technology) in 2009. Following baccalaureate education at the University of North Carolina, Dr. Rodino completed doctoral studies at Virginia Commonwealth University. Robust studies in rickettsial pathogenesis netted Dr. Rodino no less than seven primary, peer-reviewed publications (including six related to *Orientia tsutsugamuchi*) and the Mary P. Coleman Award for Extraordinary Achievement in Graduate Research. An ASM CPEP fellowship at the Mayo Clinic School of Graduate Medical Education was completed from 2018 to 2020, resulting in papers addressing alternative transport media for SARS-CoV-2 detection (18) and the clinical utility of metagenomic next-generation sequencing performed on cerebrospinal fluid specimens (19).

The ASM Award for Early Career Clinical Microbiology Research was established by the AAM in 2024 to recognize research excellence and achievements of early-career microbiologists in clinical microbiology and infectious diseases. Eligible candidates are those holding a doctoral degree (M.D., D.O., Ph.D., DrPH, Sc.D., or equivalent) and practicing in the clinical microbiology or infectious diseases field within 10 years of the completion of postdoctoral training (fellowship, residency, etc.). Suitable nominees (and awardees) are assessed on the basis of overall contributions that promote research in these fields, as evidenced by studies published in recognized scientific journals, funding awards, and scholarly work presented at national/international meetings. Candidacy can be based on a single major achievement or an aggregation of exemplary accomplishments.

In her nomination letter, Dr. Miller wrote, “Dr. Rodino exemplifies the core values of this award: innovation, leadership, scholarship, and unwavering dedication to advancing clinical microbiology. His career to date has been defined by technical excellence, intellectual curiosity, and collaborative spirit.” Dr. Rodino himself, per his personal statement, seeks to conduct research that is both practical and impactful. “In sharing this work through manuscripts and abstracts at national meetings, I hope to outline pathways for labs considering diagnostic development, guide optimal diagnostic use, promote the field, and provide important professional development opportunities for mentees.” To this end, Dr. Rodino has amassed nearly 40 peer-reviewed publications (including several in the field of metagenomics relative to SARS-CoV-2 (20) and vector-borne illnesses), ten abstracts, and over two dozen invited presentations in his provided *curriculum vitae*. He has secured substantial financial funding in either principal investigator or co-principal investigator roles.

Dr. Rodino has further distinguished himself during his early career in the leadership realm. Relative to ASM, he is active in the Eastern Pennsylvania branch of the Society, participated in the Next Generation Sequencing Working Group for ASM in 2022, and was Vice Chair of the Personnel Standards and Workforce Subcommittee in 2023 (21). In addition, Dr. Rodino served on the American Society for Clinical Pathology Awards Committee in 2023 and on the Pan American Society for Clinical Virology Annual Meeting Program Committee in 2025. A salient publication of his in 2025 addresses the potential conundrum of addressing laboratory offerings and advancement in the wake of increased enforcement discretion by the U.S. Food and Drug Administration (22).

Please join the *Journal of Clinical Microbiology* in congratulating these colleagues for their respective contributions.

## References

[1] Munson E. 2021. 2021 American Society for Microbiology awards program: clinical microbiology honorees. J Clin Microbiol 59:e00001-21. doi:10.1128/JCM.00001-21 33472897 PMC8091826

[2] Munson E. 2022. 2022 American Society for Microbiology awards program: clinical microbiology honorees. J Clin Microbiol 60:e00001-22. doi:10.1128/jcm.00001-22 PMC8091826 33472897

[3] Munson E. 2023. 2023 American Society for Microbiology awards program: clinical microbiology honorees. J Clin Microbiol 61:e00225-23. doi:10.1128/jcm.00225-23 PMC8091826 33472897

[4] Munson E. 2024. 2024 American Society for Microbiology awards and prize program: clinical microbiology honorees. J Clin Microbiol 62:e01261-24. doi:10.1128/jcm.01261-24 33472897 PMC8091826

[5] Munson E. 2025. 2025 American Society for Microbiology awards and prize program: honorees from clinical microbiology. J Clin Microbiol 63:e00001-25. doi:10.1128/jcm.00001-25 PMC7180258 31996438

[6] Burnham CD. 2025. A dazzle of zebras: launch of ASM case reports. ASM Case Rep 1:e00002-24. doi:10.1128/asmcr.00002-24 PMC12530263 41245142

[7] Munson E. 2020. 2020 American Society for Microbiology awards program honorees in clinical microbiology. J Clin Microbiol 58:e00082-20. doi:10.1128/JCM.00082-20 31996438 PMC7180258

[8] Bradbury RS, Sapp SGH, Potters I, Mathison BA, Frean J, Mewara A, Sheorey H, Tamarozzi F, Couturier MR, Chiodini P, Pritt B. 2022. Where have all the diagnostic morphological parasitologists gone? J Clin Microbiol 60:e00986-22. doi:10.1128/jcm.00986-22 36314793 PMC9667774

[9] Potters I, Bottieau E, Yansouni CP, Cnops L, Cox H, Vereecken H, Drieghe C, Verschueren J, Van den Bossche D. 2022. The case for parasitological stool microscopy. Clin Microbiol Infect 28:1310–1312. doi:10.1016/j.cmi.2022.05.031 35659926

[10] Bottieau E, Mbow M, Brosius I, Roucher C, Gueye CT, Mbodj OT, Faye BT, De Hondt A, Smekens B, Arango D, Burm C, Tsoumanis A, Paredis L, Van Herrewege Y, Potters I, Richter J, Rosanas-Urgell A, Cissé B, Mboup S, Polman K. 2024. Antimalarial artesunate-mefloquine versus praziquantel in African children with schistosomiasis: an open-label, randomized controlled trial. Nat Med 30:130–137. doi:10.1038/s41591-023-02719-4 38177851 PMC10803269

[11] Genchi M, Potters I, Kaminsky RG, Montresor A, Magnino S. 2019. Bench aids for the diagnosis of intestinal parasites. 2nd ed. World Health Organization.

[12] Potters I, Linscott AJ. 2023. Reagents, p 2721–2731. *In* Carroll KC, Pfaller MA, Karlowsky JA, Landry ML, McAdam AJ, Patel R, Pritt BS (ed), Manual of Clinical Microbiology, 13th ed. ASM Press, Washington, DC.

[13] Loeffelholz MJ, Lewinski CA, Silver SR, Purohit AP, Herman SA, Buonagurio DA, Dragon EA. 1992. Detection of Chlamydia trachomatis in endocervical specimens by polymerase chain reaction. J Clin Microbiol 30:2847–2851. doi:10.1128/jcm.30.11.2847-2851.1992 1452654 PMC270540

[14] Loeffelholz MJ, Thompson CJ, Long KS, Gilchrist MJ. 1999. Comparison of PCR, culture, and direct fluorescent-antibody testing for detection of Bordetella pertussis. J Clin Microbiol 37:2872–2876. doi:10.1128/JCM.37.9.2872-2876.1999 10449467 PMC85400

[15] Reischl U, Lehn N, Sanden GN, Loeffelholz MJ. 2001. Real-time PCR assay targeting IS481 of Bordetella pertussis and molecular basis for detecting Bordetella holmesii. J Clin Microbiol 39:1963–1966. doi:10.1128/JCM.39.5.1963-1966.2001 11326023 PMC88058

[16] Loeffelholz MJ, Samuel L, Sharp S. 2023. Laboratory accreditation and compliance, p 5–16. *In* Carroll KC, Pfaller MA, Karlowsky JA, Landry ML, McAdam AJ, Patel R, Pritt BS (ed), Manual of clinical microbiology, 13th ed. ASM Press, Washington, DC.

[17] Martin RM. 2025. 101 Topics for clinical microbiology laboratory leaders: accreditation, verification, quality systems, and more. ASM Press/Wiley, Washington, DC.

[18] Rodino KG, Espy MJ, Buckwalter SP, Walchak RC, Germer JJ, Fernholz E, Boerger A, Schuetz AN, Yao JD, Binnicker MJ. 2020. Evaluation of saline, phosphate-buffered saline, and minimum essential medium as potential alternatives to viral transport media for SARS-CoV-2 testing. J Clin Microbiol 58:e00590-20. doi:10.1128/JCM.00590-20 32229604 PMC7269412

[19] Rodino KG, Toledano M, Norgan AP, Pritt BS, Binnicker MJ, Yao JD, Aksamit AJ, Patel R. 2020. Retrospective review of clinical utility of shotgun metagenomic sequencing testing of cerebrospinal fluid from a U.S. tertiary care medical center. J Clin Microbiol 58:e01729-20. doi:10.1128/JCM.01729-20 32938739 PMC7685881

[20] Rodino KG, Peaper DR, Kelly BJ, Bushman F, Marques A, Adhikari H, Tu ZJ, Marrero Rolon R, Westblade LF, Green DA, Berry GJ, Wu F, Annavajhala MK, Uhlemann AC, Parikh BA, McMillen T, Jani K, Babady NE, Hahn AM, Koch RT, Grubaugh ND. 2022. Yale SARS-CoV-2 genomic surveillance initiative. J Clin Microbiol 60:e0060022. doi:10.1128/jcm.00600-22 35582905 PMC9199403

[21] Rodino KG, Luethy PM, Abbott AN, Bender JM, Eberly AR, Gitman M, Leber A, Dien Bard J, Personnel Standards and Workforce Subcommittee, American Society for Microbiology. 2024. Defining the value of medical microbiology consultation. J Clin Microbiol 62:e0035924. doi:10.1128/jcm.00359-24 38904385 PMC11481510

[22] Rodino KG, Larkin PMK, Graf EH. 2025. Help us to help you: recommendations for continued enforcement discretion for common infectious disease test modifications under the FDA final rule. Clin Chem 71:538–541. doi:10.1093/clinchem/hvaf018 40238134

